# Calibrated Integrated Backscatter Is Associated With Increased Left Ventricular Concentricity and Left Atrial Stiffness in Resistance Trained Individuals Using Anabolic‐Androgenic Steroids

**DOI:** 10.1111/echo.70454

**Published:** 2026-04-10

**Authors:** Florence Place, Chloe Hamilton, Harry Carpenter, Luca J. Howard, Barbara N. Morrison, Neil Chester, Robert Cooper, Ben N. Stansfield, Keith P. George, Peter Angell, David Oxborough

**Affiliations:** ^1^ Research Institute for Sport and Exercise Sciences Liverpool John Moores University Liverpool UK; ^2^ School of Human Kinetics Trinity Western University Langley British Columbia Canada; ^3^ Department of Molecular Medicine Center for Inflammation Science and Systems Medicine UF Scripps Institute for Biomedical Innovation and Technology Jupiter Florida USA; ^4^ School of Sports and Exercise Science Liverpool Hope University Liverpool UK

**Keywords:** anabolic‐androgenic steroids, calibrated integrated backscatter, cardiac remodeling, echocardiography

## Abstract

**Purpose:**

The use of Anabolic‐Androgenic Steroids (AAS) is well documented in resistance trained individuals, despite reports of myocardial inflammation and fibrosis. Calibrated integrated backscatter (ciB) is an echocardiographic technique to measure ultrasonic reflectivity of the left ventricular (LV) myocardium and is a marker for tissue characterization. This study assessed ciB in both AAS using and non‐using resistance trained individuals and documented any relationship to LV and left atrial (LA) structure and function.

**Methods:**

Male (*n* = 120) and female (*n* = 21) resistance trained individuals (age 30 ± 7 years); current (CU; *n* = 95) and non‐users (NU; *n* = 46) of AAS were recruited. Comprehensive echocardiography with strain imaging was performed. ciB was measured from a parasternal long axis and LA stiffness index (E/E′/LA reservoir strain) and left atrioventricular coupling index (LA end‐diastolic volume/LV end‐diastolic volume) were calculated.

**Results:**

ciB was significantly higher in CU (–19.54 ± 4.63) than NU (–20.88 ± 3.94, *p *= 0.047). A significant positive correlation was observed between ciB and LV mass index (r_s_(139) = 0.318, *p *< 0.001), E/E′ (r(138) = 0.169, *p *= 0.043), LA conduit strain (r(135) = –0.174, *p *= 0.043), LA stiffness index (r_s_(134) = 0.246, *p *= 0.004), and left atrioventricular coupling index (r(134) = 0.241, *p *= 0.005). ciB was not associated with any LV systolic functional parameter (global longitudinal strain or ejection fraction).

**Conclusions:**

Resistance trained individuals using AAS have significantly higher ciB than non‐users, suggesting differential myocardial tissue characteristics within this population. The presence of higher levels of ciB was significantly associated with LV geometry and LV diastolic function which may indicate a role of ciB in identifying early cardiac risk in this population.

## Graphical abstract

This study evaluates calibrated integrated backscatter (ciB) for myocardial tissue characterization in 141 resistance‐trained participants (95 AAS users, 46 non‐users). Using echocardiography, ciB was measured from the parasternal long axis orientation. Results show current AAS users have significantly higher ciB than non‐users (–19.54 vs. –20.88, *p *= 0.047). Elevated ciB significantly correlates with increased LV mass index, LA stiffness, and impaired diastolic function (e.g., left atrioventricular coupling), but not systolic function. Higher ciB levels suggest altered myocardial characteristics and may identify early cardiac risk related to AAS use.

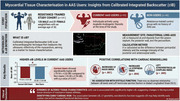

**Abbreviations**
AAS: anabolic‐androgenic steroids
BSA: body surface area
ciB: calibrated integrated backscatter
CU: current‐user
DBP: diastolic blood pressure
EF: ejection fraction
GLS: global longitudinal strain
LA: left atrium
LACI: left atrio‐ventricular coupling index
LV: left ventricle
LVEDV: left ventricular end diastolic volume
LVESV: left ventricular end systolic volume
LVM: left ventricular mass
NU: non‐user
SBP: systolic blood pressure

## 1 Introduction

Anabolic‐Androgenic Steroids (AAS) [1, 2] are widely used [3] to improve strength and perceived body image by reducing body fat and increasing muscle mass in resistance trained individuals [4]. Previous studies have demonstrated the negative cardiovascular consequences of AAS use including left ventricular (LV) hypertrophy [5, 6, 7], coronary artery disease [6], myocardial fibrosis [8] and impaired LV [9, 10], and left atrial (LA) function [11]. These findings have been explained by multiple mechanisms including atherosclerosis, thrombosis and direct myocardial injury [12]. In a rat model, AAS's exacerbated cardiac hypertrophy and was associated with upregulation of the renin angiotensin‐aldosterone system [13]. Anabolic steroid use has also been associated with an 85% increase in interstitial type I and III collagen concentration, compared to a non‐steroid group, with a concomitant decrease in LV relaxation [13]. Additionally, autopsies have demonstrated various degrees of interstitial fibrosis in AAS users [14].

Calibrated integrated backscatter (ciB) is an echocardiographic technique that quantifies the magnitude of ultrasonic reflection from tissue [15]. Early work utilizing integrated backscatter demonstrated high variability due to technical nuances and hence calibration to the reflectivity of the pericardium was proposed allowing for wider application [16]. Different tissue characteristics cause alterations in the speed and attenuation of ultrasound waves due to the tissue specific acoustic mismatch [17]. Therefore, integrated backscatter intensity, and subsequently ciB, are related, in part, to the physical and structural properties of the myocardium. For example, the extracellular matrix with its collagenous matrix, is a significant determinant of backscatter due to the marked acoustic impedance of collagen [18, 19]. In diseased populations, this technique has been applied to congestive heart failure and cardiomyopathy patients, identifying a significant positive correlation with connective tissue area [20], as well as being linked to interstitial collagen content in non‐fibrotic hearts [21]. Furthermore, in hypertrophic cardiomyopathy patients, ciB has been positively association to the magnitude of fibrosis, myocyte disarray and hypertrophy [22], and in dilated cardiomyopathy, high ciB values associate with arrhythmic burden [23]. Our group has identified a previously unknown increase in the LA stiffness index of resistance trained athletes using AAS [11]. As ciB is altered by the amount of collagen in the myocardium [21], its application in the context of evaluating LV and LA tissue characteristics in AAS users is intriguing.

The current study aims are twofold: (1) to describe ciB in resistance‐trained AAS users and non‐users and (2) to determine the association between ciB with indices of LV and LA structure and function. We hypothesized that ciB would be higher in current AAS users compared to non‐users and that increased ciB will be associated with reductions in LV and LA structure and function.

## 2 Material and Methods

### 2.1 Study Population and Design

Male (*n* = 120) and female (*n* = 21) resistance trained individuals (age 30 ± 7 years) were recruited from local gyms. Participants were grouped based on self‐reported AAS user status: current users (CU; *n* = 95) who had administered AAS during the last 12 months [24] (30 ± 67 days since last administration) and non‐users (NU; *n* = 46) who had never administered AAS. Individuals were currently undertaking a predominantly resistance training exercise regimen (>3 h per week) and had been for >2 years. Participants were excluded if they had a history of overt cardiovascular disease, diabetes, renal, or liver disease or were pregnant. Written informed consent was obtained prior to testing and ethical approval was given by the ethics committee of Liverpool John Moores University (24/SPS/017). As this project was not clinical‐based the study did not require registration as a clinical study.

The study used a cross‐sectional design whereby participants visited the lab on a single occasion. Participants completed a detailed questionnaire to capture any personal or family history of cardiovascular disease and/or medication as well as to determine specific AAS use. A 12‐lead electrocardiogram was undertaken [25] alongside a comprehensive transthoracic echocardiogram.

### 2.2 Procedures

#### 2.2.1 Participant History

A training history was obtained which included information pertaining to training experience (years), frequency (sessions per week) and duration (hours per week as well as training type). A questionnaire was used to evaluate AAS history, hence categorizing participants based on their user status. Substances used, average dose of the last month and average dose over the last year were recorded.

#### 2.2.2 Anthropometry and Examination

Anthropometric testing included an assessment of standing height (Seca Supra 719, Hannover, Germany) and body mass (Seca217, Hannover, Germany). Body surface area (BSA) was subsequently calculated using the Mosteller equation [26]. Manual brachial artery blood pressure was taken in a seated position, and systolic (SBP) and diastolic (DBP) pressures were reported.

#### 2.2.3 Echocardiography

Transthoracic echocardiography was performed by a single British Society of Echocardiography (BSE) accredited sonographer using a commercially available ultrasound system (Vivid E95, GE Healthcare, Horten, Norway) with a 1.5–4 MHz phased array transducer, with the participant lying in the left lateral decubitus position. Images were obtained according to the BSE minimum dataset [27] and the BSE guidelines for echocardiographic assessment of sports participants [28]. All examinations were acquired using the same equipment preset to include standardized settings for dynamic range, compression, and noise reduction. The parasternal long axis was optimized to ensure the septum was perpendicular to the angle of insonation. Images were stored as a raw digital imaging and communications in medicine (DICOM) format and exported to an offline analysis system (EchoPac version 202, GE Healthcare, Horton, Norway) for subsequent analysis. Both echocardiographer and investigator analyzing the data were fully blinded to user status.

#### 2.2.4 Conventional Parameters

Assessment of LV structure included calculation of LV mass (LVM) according to American Society of Echocardiography and the European Association of Cardiovascular Imaging guidelines [29]. Left ventricular end diastolic volume (LVEDV) and LV end systolic volume (LVESV) were measured in the apical four chamber and apical two chamber view using Simpsons Biplane. Left ventricular mass and volumes were scaled ratiometrically or allometrically to account for geometric similarity [30]. Left ventricular mass was determined using the American Society of Echocardiography corrected equation [29]. Concentricity was calculated as LVM/LVEDV^0.667^ [31]. Ejection fraction (EF) was calculated using the Simpson's biplane method. Left atrioventricular coupling index (LACI) was calculated as LA end‐diastolic volume/LV end‐diastolic volume.

An assessment of LV diastolic function included pulsed wave Doppler of transmitral flow during early (E) and late (A) diastole, and E/A ratio was calculated. Tissue Doppler Imaging allowed for the assessment of mitral annular early diastolic (E’) velocity in the apical four‐chamber view for the septum and lateral walls, and an average E’ and E/E’ were calculated.

#### 2.2.5 Two‐Dimensional Myocardial Speckle Tracking Echocardiography

Speckle tracking echocardiography was utilized for assessment of LV (GLS) and LA longitudinal strain. Images were optimized to ensure optimal spatial resolution with frame rates of 40–90 FPS. Offline analysis of strain was made in accordance with current guidelines [32, 33]. A single cardiac cycle was used, and zero strain reference was set at ventricular end diastole for both the LV and LA. The region of interest (LV and LA myocardium) was traced and divided into six equidistant segments from each view and were subsequently tracked throughout the cardiac cycle. Global LA strain during the reservoir, conduit and booster phases were measured with LA stiffness index calculated as the ratio of E/E’ to LA reservoir strain [34].

#### 2.2.6 Calibrated Integrated Backscatter

**FIGURE 1:**
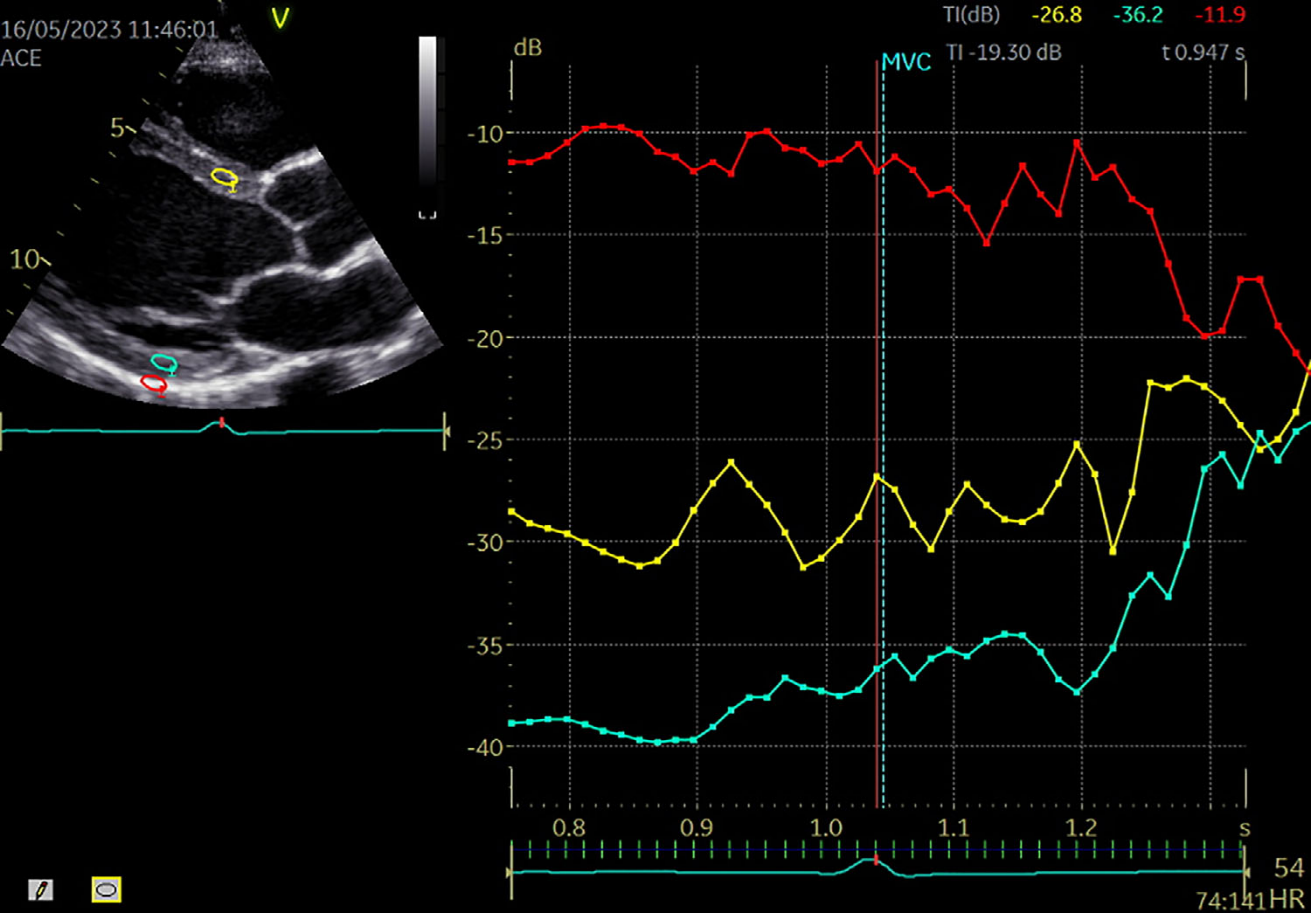
Integrated backscatter assessment of anteroseptal wall (yellow), posterior wall (blue) and pericardium (red) [52].

Left ventricular ciB was measured from a parasternal long‐axis orientation. A sample area (4 × 8 mm) was placed in the mid‐myocardium of the antero‐septum, posterior wall and pericardium at end‐diastole with care taken to exclude endocardial and epicardial tissue (see Figure 1). The ciB was calculated by subtracting pericardial integrated backscatter intensity from the average intensity of the posterior and anteroseptal walls and averaged over two cardiac cycles.

### 2.3 Statistical Analysis

Statistical analyses were performed using the commercially available software package IBM SPSS Statistics for Mac, Version 29.0.2.0 (IBM, Illinois, USA). All indices were assessed for normality using a Kolmogorov–Smirnov test and are presented as mean ± standard deviation (SD) or median [95% confidence intervals (CI)] as appropriate. Differences between current and non‐users were analyzed using an independent *t*‐test or Mann‐Whitney U test as appropriate with significance accepted at *p *< 0.05. Associations between ciB and structural and functional parameters were assessed using Pearson's correlation or Spearman's rank correlation depending on the distribution of the data. In a random subsample of 10 participants, ciB was independently measured by a second observer. Interobserver reliability was assessed using the intraclass correlation coefficient (ICC) and the coefficient of variation (CoV) [35].

## 3 Results

### 3.1 Participant Demographics and Image and Performance Enhancing Drug Use

The types of performance enhancing drugs used by CU are presented in Table 1. All were using AAS. Current users had a history of use of 7 ± 5 years, were taking 5 ± 3 substances simultaneously, mean dose over the previous month was 1527 ± 1497 mg·wk^−1^ [0 – 2196 mg·wk^−1^] and mean dose over the previous year was 879 ± 1210 mg·wk^−1^ [19 – 7265 mg·wk^−1^]. Anthropometric and training characteristics are presented in Table 2. Training duration (years) was similar between groups, but CU presented with greater training hours per week than NU (*p* < 0.001). Body mass (*p* < 0.001), height (*p* = 0.025), BSA (*p* < 0.001), SBP (*p *= 0.016), and heart rate (*p* = 0.004) were greater in CU compared to NU. There was no difference in DBP between CU and NU (*p* > 0.05).

**TABLE 1:** Image and performance enhancing drug use.

Current users (*n* = 95)	
Anabolic‐androgenic steroids	95
Beta 2 agonist	14
Growth hormones and growth hormone secretagogues/agonists	32
Insulin	14
Metformin	10
Aromatase inhibitors/anti‐estrogens	4
Thyroid hormones	18
Diuretics	1

**TABLE 2:** Anthropometric and training characteristics.

Variable	Current user Mean ± SD or Median [95% CI]	Non‐user Mean ± SD or Median [95% CI]	*p* value
Sample size	95	46	
Age (years)	31 [30, 33]	24 [25, 29]	<0.001
Weight (kg)	99 ± 17	79 ± 17	<0.001
Height (cm)	177 ± 8	173 ± 9	0.014
BSA (m^2^)	2.20 ± 0.23	1.94 ± 0.24	<0.001
Heart rate (bpm)	66 ± 11	59 ± 10	<0.001
Systolic blood pressure (mmHg)	128 ± 11	124 ± 13	0.016
Diastolic blood pressure (mmHg)	72 ± 9	74 ± 9	0.202
Training duration (years)	11 [11, 15]	12 [10, 15]	0.427
Training hours (per week)	10 [10, 12]	8 [7, 9]	<0.001

Abbreviation: BSA, body surface area.

### 3.2 Echocardiographic Data

#### 3.2.1 Left Ventricular Structure and Function

Left ventricular parameters are demonstrated in Table 3. Current users had significantly greater LVEDV (U = 775, *p *< 0.001), LVEDV index (U = 1484, *p *= 0.004), LVESV (U = 669, *p *< 0.001), LVESV index (U = 1088, *p *< 0.001), LVM (U = 382, *p *< 0.001), LVM index (U = 426, *p* < 0.001), concentricity (U = 497, *p *< 0.001), transmitral A wave (U = 1536, *p *= 0.004), and E/E’ (U = 1160, *p *< 0.001) compared to NU. E/A ratio (U = 1404, *p* < 0.001), GLS (t(133) = 8.37, *p *< 0.001), and EF (U = 1180, *p *< 0.001) were significantly lower in CU.

**TABLE 3:** Left ventricular structure and function.

Variable	Current user Mean ± SD or Median [95% CI]	Non‐user Mean ± SD or Median [95% CI]	*p* value
LVEDV (mL)	161 [157, 173]	121 [116, 131]	<0.001
LVEDV index (mL·m^−2^)	50 [49, 52]	47 [44, 48]	0.004
LVESV (mL)	72 [71, 80]	50 [47, 54]	<0.001
LVESV index (mL·m^−2^)	22 [22, 24]	19 [18, 20]	<0.001
LV Mass (g)	241 [237, 275]	140 [135, 154]	<0.001
LV Mass Index (g·m^−2^)	110 [108, 121]	73 [71, 78]	<0.001
E wave (m·s^−1^)	0.79 ± 0.14	0.82 ± 0.16	0.083
A wave (m·s^−1^)	0.52 [0.52, 0.56]	0.47 [0.44, 0.51]	0.004
E/A ratio	1.5 [1.4, 1.6]	1.8 [1.7, 2.0]	<0.001
Average E/E’	6.6 [6.5, 7.2]	5.4 [5.3, 6.1]	<0.001
Concentricity (g·mL^−0.667^)	8.0 [8.0, 8.8]	5.7 [5.5, 6.2]	<0.001
GLS (%)	−15.1 ± 2.1	−18.1 ± 1.7	<0.001
EF (%)	55 ± 6	59 ± 4	<0.001

Abbreviations: BSA, body surface area; EF, ejection fraction; GLS, global longitudinal strain; LV, left ventricular; LVEDV, left ventricular end‐diastolic volume; LVESV, left ventricular end‐systolic volume.

#### 3.2.2 Left Atrial Structure and Function

Left atrial parameters are shown in Table 4. Current users had significantly lower LA reservoir strain (t(135) = –6.03, *p *< 0.001) and conduit strain (t(135) = –6.33, *p *< 0.001) compared to NU. Left atrial stiffness index (U = 713, *p *< 0.001), LA end‐systolic volume index (LAVi; t(135) = 2.13, *p *= 0.035) and LACI (t(134) = –2.02, *p *= 0.045) were significantly greater in CU than NU.

**TABLE 4:** Left atrial structure and function.

Variable	Current user Mean ± SD or Median [95% CI]	Non‐user Mean ± SD or Median [95% CI]	*p* value
LA reservoir strain (%)	32 ± 7	40 ± 7	<0.001
LA conduit strain (%)	21 ± 6	28 ± 6	<0.001
LA booster strain (%)	13 ± 4	14 ± 4	0.300
LA stiffness index	0.21 [0.20, 0.24]	0.14 [0.13, 0.16]	<0.001
LA end systolic volume index (mL·m^−2^)	18 ± 4	17 ± 4	0.035
LACI	0.14 ± 0.05	0.12 ± 0.04	0.045

Abbreviations: LA, left atrial; LACI, left atrioventricular coupling index.

#### 3.2.3 Calibrated Integrated Backscatter

**FIGURE 2:**
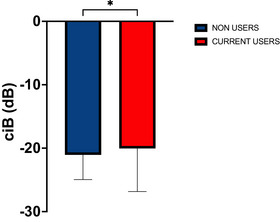
Calibrated integrated backscatter (ciB). * Denotes significance *p *< 0.05.

Calibrated Integrated Backscatter was significantly higher in CU (–19.54 ± 4.63 dB) compared to NU (–20.88 ± 3.94 dB, t(139) = 1.69, *p *= 0.047; see Figure 2).

#### 3.2.4 Relationship Between ciB and Cardiac Parameters

**FIGURE 3:**
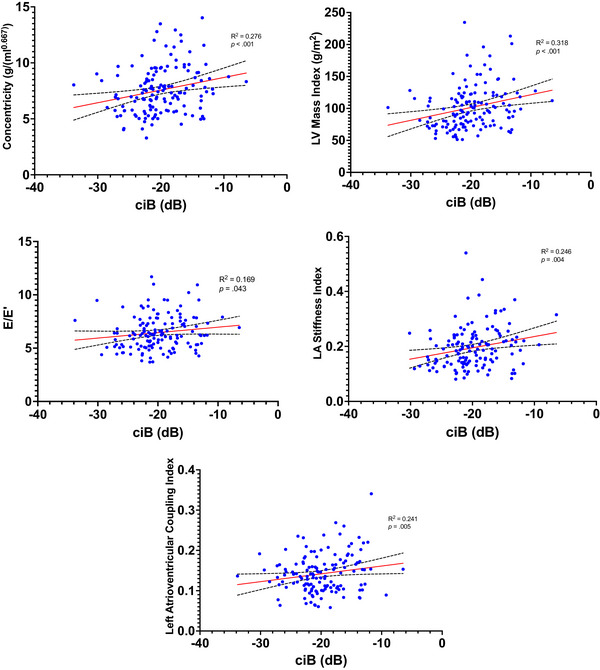
The relationship between calibrated integrated backscatter and cardiac parameters.

A significant positive correlation was observed between ciB and concentricity (r_s_(138) = 0.276, *p *< 0.001), LVMi (r_s_(139) = 0.318, *p *< 0.001), E/E’ (r(138) = 0.169, *p *= 0.043), LA conduit strain (r(135) = –0.174, *p *= 0.043), LA stiffness index (r_s_(134) = 0.246, *p *= 0.004), LAVi (r(136) = 0.203, *p *= 0.018), and LACI (r(134) = 0.241, *p *= 0.005; see Figure 3A–E). No relationship was observed between ciB and GLS (r(133) = 0.089, *p *= 0.306), EF (r_s_(138) = –0.103, *p *= 0.226), or LA reservoir strain (r(135) = –0.161, *p *= 0.060).

Interobserver reliability for ciB was good, with an intraclass correlation coefficient ICC of 0.86 (95% CI: 0.40–0.97, *p* < 0.001). The mean CoV was 7%.

## 4 Discussion

To the best of our knowledge this is the first study to explore ciB and its association with LV and LA structure and function in AAS users. The main findings were: (1) resistance trained AAS users had higher ciB than non‐users and (2) ciB was significantly associated with E/E’, LA conduit strain and LA stiffness index, viewed as “early” measures of cardiac dysfunction.

Calibrated integrated backscatter is an ultrasound technique that quantifies the magnitude of ultrasonic reflection from tissue [15] and hence provides a measure of LV myocardial tissue characterization [36]. As hypothesized, the current study demonstrated a higher ciB in AAS users compared to non‐users, suggesting differential myocardial tissue characteristics within this population. Supraphysiological doses of AAS have been shown to induce cardiotoxicity with proposed mechanisms including myocyte hypertrophy, disarray, and fibrosis [37] identified through biopsy and corroborated by histological assessment of post‐mortem tissue [8, 38]. In an animal study, exercise training rats treated with AAS demonstrated an increase in angiotensin converting enzyme and type III collagen expression, compared to non‐AAS using exercise matched controls [13]. Therefore, although the mechanisms of AAS‐induced changes in cardiac structure are beyond the scope of this paper, it is reasonable to suggest that changes in cardiac tissue characteristics (increased collagen content and myocyte disarray) may contribute to differences in ciB observed in this study. Supportively, ciB has been shown to be associated with total and interstitial myocardial fibrosis due to the increased collagen content of fibrotic tissue [20, 21]. Additionally, ciB has been significantly positively correlated with the extent of histologically assessed myocyte disarray [22], suggestively due to increased ultrasound scattering from disorganized myocyte orientation [39].

The relationship between ciB and altered tissue characteristics has previously been demonstrated in hypertrophic cardiomyopathy [16, 22], the pathology of which has been likened to that of AAS‐induced cardiac changes [2]. In a study investigating ciB in hypertrophic cardiomyopathy patients [22], septal (–23.9 ± 2.9 dB vs. –30 ± 0.7 dB, *p *< 0.05) and posterior (–24.6 ± 1.4 dB vs. –32 ± 2.7 dB, *p* < 0.05) wall, ciB were higher in hypertrophic cardiomyopathy patients compared to a healthy control group. Additionally, in a study of metabolic syndrome patients [40], posterior wall ciB was higher in metabolic syndrome patients than in healthy controls (−24.2 ± 5.4 dB vs. −25.3 ± 5.8 dB, *p *< 0.001). Notably, septal ciB was significantly higher in participants with “high” degree of fibrosis compared to “low” degree of fibrosis (*p *< 0.05), classified based on levels of procollagen peptides [40]. Within the population described in our study, we observed higher ciB values than in these clinical populations. These studies had older study populations than the current study; thus, age‐related changes in tissue characteristics may explain these differences. Additionally, similar to other cardiac parameters (e.g., coronary atherosclerotic burden [6]), the changes in myocardial tissue characteristics may be dependent on dose and duration of use of AAS, of which there was wide variation within this population (dose = 1527 ± 1497 mg·wk^−1^, duration of use = 6 ± 5 years).

Notably, ciB was associated with early measures of cardiac dysfunction, including E/E’, LA conduit strain and LA stiffness index. Reductions in LA strain (conduit and reservoir) may occur prior to parameters conventionally used to diagnose diastolic dysfunction (lateral and septal E’) [41] due to its sensitivity to LV end‐diastolic [42] and LA pressures [43]. Additionally, LA reservoir and conduit strain have been found to be reduced in current users compared to non‐users of AAS, likely due to the cardiotoxic effects of AAS alongside the impact of LV diastolic dysfunction [11]. Furthermore, E/E’ has been found to be higher in current users compared to non‐users of AAS [9, 44], indicative of reduced ventricular diastolic function and increased LV filling pressures [45]. Taken together, these findings may illustrate the presence of subclinical impairment in LV diastolic function in this population. Left ventricular diastolic dysfunction is linked to stiffer myocardial muscle [36] and a significant relationship has been found between the degree of myocyte disarray and diastolic dysfunction in hypertrophic cardiomyopathy patients [46]. Supportively, Kobayashi et al. [47] found an inverse relationship between histopathologically assessed severity of myocyte disarray and septal systolic and early diastolic strain rate, attributed to the impact of AAS use on myocyte disarray [37]. It has also been suggested that changes in myocardial collagen isoform expression (type I to type III ratio), may play a role in impairments of diastolic function [48, 49], and are increased in an animal model of AAS users [13]. As these structural alterations (i.e., myocyte disarray and increased collagen content) can promote LV hypertrophy [50], it is notable that ciB was positively correlated with both LVM index and concentricity, but not with measures of systolic function (EF and GLS), supporting the suggestion that increasing ciB in this cohort is more closely related to structural myocardial remodeling than to systolic function. As ciB is a measure of tissue characterization, these findings outline the possible mechanisms linking E/E’, LA strain, LA stiffness, and LV geometry with ciB. Clinically, ciB may be used in early investigations to determine subclinical myocardial changes in AAS users who may warrant closer follow‐up and could be used alongside standard echocardiography. Potential earlier identification of cardiac risk in AAS users can guide individual intervention as well as public health messaging.

## 5 Limitations

Despite our findings, it is important to note some limitations of this study. With respect to ciB, there are no published reference values, which challenge clinical interpretation between groups and studies. We presented data from a large cohort of self‐report AAS users. Whilst our research group is well embedded in the athlete group and culture to promote honesty and accuracy of reporting, we cannot rule out bias, recall error and other facets of self‐reported data. The participants were very open regarding their AAS use and were aware of the study aims. We also note that self‐report has been found to be highly sensitive and specific in the detection of urinary AAS [51]. Due to the study design and recruitment processes (self‐selection), participants were heterogeneous with regard to age, weekly dose, and body composition. These factors are likely to confound the data with a strong potential for co‐linearity. In view of this further repeated measures and/or longitudinal studies should be undertaken to establish the direct cardiac impact of AAS use with regard to cumulative exposure, acute effects, cessation of use, and dose response. Future work should also aim to capture more detailed information on the substances used, including specific compounds alongside biochemical confirmation. Furthermore, although myocyte disarray, collagen, and fibrosis content have been speculated as potential mechanisms for the between‐group differences found within this study, these were not directly measured, and therefore, future studies should aim to assess the prevalence within this population utilizing cardiac magnetic resonance. Although interobserver variability was acceptable (CoV = 7%), this exceeded the absolute difference observed between groups. While a statistically significant difference was still detected, this degree of measurement error may limit the clinical applicability of the findings at this stage.

## 6 Conclusions

In conclusion, resistance trained AAS users have a significantly higher ciB than non‐users, suggesting differences in myocardial tissue characteristics. Higher ciB was significantly associated with markers of diastolic dysfunction, which may indicate a role for ciB in identifying early cardiac risk in this population.

## Conflicts of Interest

The authors declare no conflicts of interest.

## Funding

The authors have nothing to report.
